# Results from Genetic Studies in Patients Affected with Craniosynostosis: Clinical and Molecular Aspects

**DOI:** 10.3389/fmolb.2022.865494

**Published:** 2022-04-28

**Authors:** Ewelina Bukowska-Olech, Anna Sowińska-Seidler, Dawid Larysz, Paweł Gawliński, Grzegorz Koczyk, Delfina Popiel, Lidia Gurba-Bryśkiewicz, Anna Materna-Kiryluk, Zuzanna Adamek, Aleksandra Szczepankiewicz, Paweł Dominiak, Filip Glista, Karolina Matuszewska, Aleksander Jamsheer

**Affiliations:** ^1^ Department of Medical Genetics, Poznan University of Medical Sciences, Poznan, Poland; ^2^ Department of Head and Neck Surgery for Children and Adolescents, University of Warmia and Mazury in Olsztyn, Olsztyn, Poland; ^3^ Prof. St. Popowski Regional Specialized Children's Hospital, Olsztyn, Poland; ^4^ Department of Medical Genetics, Institute of Mother and Child, Warsaw, Poland; ^5^ Centers for Medical Genetics GENESIS, Poznan, Poland; ^6^ Biometry and Bioinformatics Team, Institute of Plant Genetics, Polish Academy of Sciences, Poznan, Poland; ^7^ Celon Pharma S.A., Medicinal Chemistry Department, Lomianki, Poland; ^8^ Poznan University of Medical Sciences, Poznan, Poland; ^9^ Molecular and Cell Biology Unit, Department of Paediatric Pulmonology, Allergy and Clinical Immunology, Poznan University of Medical Sciences, Poznan, Poland

**Keywords:** calvarial sutures, craniosynostosis, next-generation sequencing, chromosomal microarray analysis, cohort screening

## Abstract

**Background:** Craniosynostosis (CS) represents a highly heterogeneous genetic condition whose genetic background has not been yet revealed. The abnormality occurs either in isolated form or syndromic, as an element of hundreds of different inborn syndromes. Consequently, CS may often represent a challenging diagnostic issue.

**Methods:** We investigated a three-tiered approach (karyotyping, Sanger sequencing, followed by custom gene panel/chromosomal microarray analysis, and exome sequencing), coupled with prioritization of variants based on dysmorphological assessment and description in terms of human phenotype ontology. In addition, we have also performed a statistical analysis of the obtained clinical data using the nonparametric test χ^2^.

**Results:** We achieved a 43% diagnostic success rate and have demonstrated the complexity of mutations’ type harbored by the patients, which were either chromosomal aberrations, copy number variations, or point mutations. The majority of pathogenic variants were found in the well-known CS genes, however, variants found in genes associated with chromatinopathies or RASopathies are of particular interest.

**Conclusion:** We have critically summarized and then optimised a cost-effective diagnostic algorithm, which may be helpful in a daily diagnostic routine and future clinical research of various CS types. Moreover, we have pinpointed the possible underestimated co-occurrence of CS and intellectual disability, suggesting it may be overlooked when intellectual disability constitutes a primary clinical complaint. On the other hand, in any case of already detected syndromic CS and intellectual disability, the possible occurrence of clinical features suggestive for chromatinopathies or RASopathies should also be considered.

## Introduction

Premature fusion of calvarial sutures, i.e., craniosynostosis (CS), represents a highly heterogeneous neurocranium malformation (Morriss-Kay and Wilkie, 2005). The disease can be classified following at least three criteria: the origin, the number of affected sutures, and the occurrence of additional clinical features. Regarding the etiology, CS is distinguished as a primary condition, i.e., genetic; or secondary, if it arises from mechanical, metabolic, or hormonal defects. The assessment of affected sutures’ numbers allows recognising single or compound CS, and finally, a syndromic CS is described when additional features other than secondary to premature suture fusion occur. Otherwise, CS should be classified as an isolated condition (Wilkie et al., 2007; Johnson and Wilkie, 2011; Lattanzi et al., 2017).

The neonatal skull comprises six calvarial sutures—one metopic, one sagittal, two coronal, and two lambdoid, closing physiologically from 3 months to late 50 years of postnatal life. Calvarial sutures are fibrous junctions, which allow the skull to grow and develop during the expansion of the brain and permit skull compression during delivery (Baer, 1954; Opperman, 2000; Rice, 2008). Thus, the lack of physiological sutural patency impedes the allometric cranial growth, resulting in cranial deformities and, frequently, increased intracranial pressure, i.e., craniostenosis. Consequently, affected patients present with facial dysmorphism, cortex lesion, seizures, intellectual disability, visual and hearing impairments, or breathing difficulties that are all secondary to CS (Renier et al., 1982; Thompson et al., 1995; Tubbs et al., 2001; Gupta et al., 2003; Mathijssen and Arnaud, 2007; Chieffo et al., 2010).

CS affects approximately one in 2,500 births and burdens public health due to the requirement of extensive surgical treatment in the first year of life and multi-level specialist medical care in the subsequent postnatal periods (Wilkie et al., 2010; Lattanzi et al., 2017). Despite recent advancements in genetic diagnostics, the pathogenesis of CS remains still unknown or partially understood. The large cohort screenings reveal genetic etiology in barely 21–62% of all recruited cases, depending on the size of the study, ethnicity of the population, and range of the molecular analysis. Conversely, about 40–80% of CS cases remain molecularly unresolved (Roscioli et al., 2013; Paumard-Hernandez et al., 2015; Timberlake et al., 2016; Lee et al., 2018; Topa et al., 2020). In this paper, we have presented the study results encompassing 166 individuals in whom we had applied a three-step diagnostic algorithm to identify different mutation types. In addition, we have also performed a statistical analysis of the clinical data we had obtained.

## Patients and Methods

All procedures involving human participants were performed under the Helsinki Declaration. The Institutional Review Board of Poznan University of Medical Sciences granted ethics approval (no. 742/17). All patients agreed to participate in this study, and written informed consent for participation and publishing the information and images in an online open-access publication was obtained from all participants and the parents of minors before genetic testing.

### Cohort Description

The patients were recruited provided they were born from pregnancies without exposure to environmental factors potentially causative for CS. Our cohort consisted of 166 individuals (33 patients belonging to 18 families and 133 sporadic patients), of whom 85 were males and 81 females. All patients underwent dysmorphological assessment, which allowed us to section off the two subgroups—isolated (if CS was accompanied only by the secondary defects directly resulting from CS) and syndromic (if CS was accompanied by additional defects, not resulting from CS).

### Statistical Analysis

STATISTICA (version 13.3) TIBCO software was used for data analysis. The statistical significance of the phenotypic diversity of the cohort and the differences between the frequency of identified genetic modification in the different phenotypic groups was tested using the nonparametric test χ^2^.

### Genetic Studies

We extracted genomic DNA from peripheral blood leukocytes drawn into EDTA-coated tubes using either the manual salting-out method or automated extraction using the MagCore^®^ HF16 Automated Nucleic Acid Extractor (RBC Bioscience Corp.). Whole blood for lymphocyte culture was drawn into heparin-coated tubes. The study has been divided into three tiers. Tier 1 included karyotyping and screening of the most frequent mutations located in exon no. 7 of *FGFR1* (NM_023110.3), exons no. 7 and 8 of *FGFR2* (NM_000141.5), and exon no. 7 of *FGFR3* (NM_000142.5), and the entire coding sequence of *TWIST1* (NM_000474.4)*.* In tier 2, chromosomal microarray analysis and targeted next-generation sequencing (NGS) of custom gene panel were performed. Exome sequencing (ES) was applied in Tier 3.

### Tier 1

#### PCR and Sanger Sequencing

We performed molecular screening for all recruited patients (n = 166) utilizing targeted PCR followed by Sanger sequencing. We tested the occurrence of the most frequent, recurrent mutations located in exon 7 of *FGFR1* (NM_023110.3), exons 7 and 8 of *FGFR2* (NM_000141.5), and exon 7 of *FGFR3* (NM_000142.5), and the entire coding sequence of *TWIST1* (NM_000474.4)*.* Specific primers for amplification were designed using the online available Primer3 tool v. 0.4.0. For the detailed list of primers, see Supplementary Table S1. PCR products were sequenced using dye-terminator chemistry (kit v.3, ABI 3130XL) and run on automated sequencer Applied Biosystems Prism 3700 DNA Analyzer.

#### Karyotyping

Whole blood lymphocyte culture was performed following the standard protocol. Next, we used the Giemsa-banding (GTG) technique at 550 band resolution per haploid genome.

### Tier 2

#### Chromosomal Microarray Analysis

Patients suspected of harboring pathogenic copy number variations (CNVs) were tested using (CMA). Depending on the research stages’, different CMA formats were used. Firstly, the assay was performed employing a high resolution 1.4 NimbleGen oligonucleotide array comparative genomic hybridization (aCGH; Roche NimbleGen) according to standard protocols provided by the manufacturer. Results were analysed with Deva software (Roche Nimblegen) using the ADM2 segmentation algorithm (Sowińska-Seidler et al., 2018). The chromosomal profile was visualized using SignalMap software (NimbleGen Systems Inc.). Next, we applied SurePrint G3 Human CGH Microarray 8 × 60 k, 4 × 180 k, 1 × 1M arrays (Agilent Technologies). The hybridization signals were detected with SureScan Dx Microarray Scanner (Agilent Technologies) and visualized with the use of Agilent CytoGenomics software (Agilent Technologies) (Bukowska-Olech et al., 2020b). The pathogenicity of CNVs was evaluated using the following tools and available online databases-Cytoscape 3.7.1, ClinGen, DECIPHER, database of Genomic Variants (DGV), Mouse Genome Informatics (MGI), or UCSC Genome Browser applying tracks such as Conservation, VistaEnhancers, ENCODE Regulation or HiC.

#### NGS of Custom Gene Panel

A cohort with negative results was subjected to targeted next-generation sequencing of a custom 225.709 kb in size gene panel (Agilent Technologies). Captured and indexed libraries were sequenced on the previously described Ion Torrent S5 sequencing system. Variants identified by TorrentSuite, as first described by Bukowska-Olech et al., were further analyzed using an extended custom pipeline (Bukowska-Olech et al., 2020c). For prioritizing candidate variants in probably causative genes, local Phen2Gene installation was used. For final SNV/indel prioritization, the updated pipeline combined Exomiser 12.1.0 with ANNOVAR (all non-commercially available databases, downloaded on 16th December 2020), Ensembl/VEP 102.0, and CADD 1.6 Exomiser default phenotype scoring was supplemented with alternate scoring where the original formula used Phen2Gene scores instead (Zhao et al., 2020). Two prioritizers (OMIM and HIPHIVE) were used with Exomiser. For both Phen2Gene and Exomiser, each sample was labeled with Human Phenotype Ontology terms assigned according to clinical notes (manual curation) (Supplementary Table S2). Common (AF>0.001 in population frequency datasets) and benign (as per strong ClinVar support) alleles were dropped out for reporting. The final pathogenicity of detected variants was analysed in line with the American College of Medical Genetics (ACMG) classification (Richards et al., 2015). Confirmation and segregation studies were performed applying PCR followed by Sanger sequencing as described in section *PCR and Sanger sequencing*. A list of primers was summarized in Supplementary Table S1.

### Tier 3

#### Exome Sequencing

The coding region and flanking intronic regions were enriched using a custom-designed in-solution exome enrichment (TWIST bioscience, San Francisco, United States) and were sequenced using the Illumina NovaSeq system (Illumina, San Diego, United States). Sequencing reads were demultiplexed using Illumina bcl2fastq2. The removal of the adapter was performed with Skewer. The trimmed reads were mapped to the human reference genome (hg19) using the Burrows-Wheeler Aligner, and variants were called using in-house software. First, Only SNVs and small indels in the coding regions and the flanking intronic regions (±8 bp) with a minor allele frequency (MAF) < 1.5% were evaluated. Second, the known disease-causing variants (according to Human Gene Mutation database; HGMD) were also evaluated in up to ±30 bp of flanking regions and up to 5% MAF. Downstream analysis was carried out using pipeline described for Tier 2 above. As before, the variant evaluation was based on the ACMG guidelines for interpreting sequence variants. Confirmation and segregation studies were performed applying PCR followed by Sanger sequencing as described in section *PCR and Sanger sequencing*. A list of primers was summarized in Supplementary Table S1.

## Results

We summarized all differentiating phenotypic features in our cohort in Table 1 and Supplementary Table S3. Patients involved in this study were more frequently sporadic (81%) than familial cases (19%)–χ^2^ (1;166) = 62.67; *p* < 0.001. We have not revealed any differences between the occurrence of isolated (56%) and syndromic forms of CS (44%)–χ^2^ (1; 146) = 2.22; *p* = 0.14. However, we have reported more frequently single CS (66%) than multiple CS (34%)–χ^2^ (1;144) = 14.69; *p* < 0.001. Next, we have also shown that the most often affected suture in isolated CS was coronal (53%), followed by metopic (33%), sagittal (15%), and lambdoid (0%)–χ^2^ (3;95) = 51.54; *p* < 0.001.

**TABLE 1:** The phenotypic characterization of the cohort of 166 patients affected with craniosynostosis.

Sex	Frequency (%)
Female	49
Male	51
**Total**	100
**Number of affected sutures**	**Frequency (%)**
Multiple	34
Single	66
metopic	25
coronal	53
sagittal	22
lambdoid	0
**Total**	100
**Occurence**	**Frequency (%)**
Familial	19
Sporadic	81
**Total**	100

Our diagnostic success was 43% (n = 72). The following paragraphs described the detailed results, entitled Tier 1–3, which were also summarized in Tables 2–4. Mutations located within the *FGFR1*, *FGFR2*, *FGFR3* genes, and in the *TWIST1* gene constitute the most often occurring alterations (73%)–χ^2^ (1;71) = 14.43; *p* < 0.001. This diagnostic indicator occurred significantly statistic (χ2 (1;52) = 4.92; *p* < 0.05) more often in a female group of patients (48%, *FGFR2* was the most frequently affected–19%) than male–25% (the most common pathogenic variants were located in the *TWIST1* gene–12%).

Regarding sex, we have not revealed any relevant diagnostic success rate changes, which were made in 62% among female patients and 38% among male patients–χ^2^ (1;71) = 3.61; *p* = 0.06. Next, we have shown that more often, we could diagnose the patients affected with multiple CS (65%) than single CS (35%)–χ^2^ (1;49) = 4.50; *p* = 0.03. Detailed results were summarized in Supplementary Table S4.

### Tier 1

PCR followed by Sanger sequencing of the most frequent mutations located within the *FGFR1*, *FGFR2*, *FGFR3* genes and screening of the entire *TWIST1* coding sequence allowed us to diagnose 43 patients from 32 different families. Out of them, three patients carried the same alteration in the 7^th^ exon of *FGFR1* gene, 15 individuals presented with one from 10 mutations in the *FGFR2* gene, 10 patients harbored one recurrent variant in the *FGFR3*, whereas 15 patients harbored nine distinct variants in the *TWIST1* gene (Table 2). Karyotyping revealed three heterozygous deletions: one in locus 7q32.3-q35, second in locus 9p, and third in locus 18q21.32-q23 (Supplementary Table S5). The first two were additionally resized using 4 × 180 k Agilent CMA, while the third was by ES.

**TABLE 2:** The list of point variants found in the cohort of 166 patients affected with craniosynostosis. HGMD, Human Gene Mutation database (accession date: October 2021); B, bilateral; C, coronal; I, isolated; L, lambdoid; M, metopic; SA, sagittal; U, unilateral.

#	Patient ID	Sex	Gene	Reference sequence	Genomic Location (GRCh38)	coding DNA	Protein	HGMD	Affected suture(s)	Type of CS
1	P94	M	*ALX3*	NM_006492.3	Chr1:110064600-110064603del	c.578_581del	p.Thr193Argfs*137	-	CU	I
2	P134	M	*ARID1A*	NM_006015.6	Chr1:26697194C>A	c.791C>A	p.Ser264*	-	M, SA	S
3	P91	F	*EFTUD2*	NM_004247.4	Chr17:44883094T>C	c.491A>G	p.Asp164Gly	CM2018685	M	S
4	P132	M	*ERF*	NM_001301035.1	Chr19:42249493G>A	c.394C>T	p.Arg132*	-	SA, LU	S
5	P136	F	*FAM111A*	NM_022074.4	Chr11:59152581A>G	c.913A>G	p.Arg305Gly	-	CB, LB	S
6	P21	M	*FGFR1*	NM_023110.3	Chr8:38424690G>C	c.755C>G	p.Pro252Arg	CM940776	N/A	N/A
7	P22	M	*FGFR1*	NM_023110.3	Chr8:38424690G>C	c.755C>G	p.Pro252Arg	CM940776	N/A	N/A
8	P23	F	*FGFR1*	NM_023110.3	Chr8:38424690G>C	c.755C>G	p.Pro252Arg	CM940776	N/A	N/A
9	P25	F	*FGFR2*	NM_000141.5	Chr10:121520163G>C	c.755C>G	p.Ser252Trp	CM950458	N/A	N/A
10	P31	F	*FGFR2*	NM_000141.5	Chr10:121520163G>C	c.755C>G	p.Ser252Trp	CM950458	N/A	N/A
11	P32	F	*FGFR2*	NM_000141.5	Chr10:121520163G>C	c.755C>G	p.Ser252Trp	CM950458	N/A	N/A
12	P35	M	*FGFR2*	NM_000141.5	Chr10:121520163G>C	c.755C>G	p.Ser252Trp	CM950458	N/A	N/A
13	P29	F	*FGFR2*	NM_000141.5	Chr10:121520160G>C	c.758C>G	p.Pro253Arg	CM950459	CB	S
14	P30	F	*FGFR2*	NM_000141.5	Chr10:121520160G>C	c.758C>G	p.Pro253Arg	CM950459	N/A	N/A
15	P33	F	*FGFR2*	NM_000141.5	Chr10:121520160G>C	c.758C>G	p.Pro253Arg	CM950459	CB, LU	N/A
16	P34	F	*FGFR2*	NM_000141.5	Chr10:121520160G>C	c.758C>G	p.Pro253Arg	CM950459	N/A	N/A
17	P27	M	*FGFR2*	NM_000141.5	Chr10:121520076T>C	c.842A>G	p.Tyr281Cys	CM013715	N/A	N/A
18	P12	F	*FGFR2*	NM_000141.5	Chr10:121520052T>G	c.866A>C	p.Gln289Pro	CM950462	CB, SA	S
19	P47	M	*FGFR2*	NM_000141.5	Chr10:121520050A>C	c.868T>G	p.Trp290Gly	CM1313533	S	S
20	P24	M	*FGFR2*	NM_000141.5	Chr10:121517445T>C	c.958A>G	p.Thr320Ala	CM1919088	N/A	I
21	P26	F	*FGFR2*	NM_000141.5	Chr10:121517411T>A	c.992A>T	p.Asn331Ile	CM960645	N/A	N/A
22	P5	M	*FGFR2*	NM_000141.5	Chr10:121517378C>T	c.1025G>A	p.Cys342Tyr	CM940779	CB, SA	I
23	P65	F	*FGFR2*	NM_000141.5	Chr10:121517378C>T	c.1025G>A	p.Cys342Tyr	CM940779	M, S	I
24	P6	F	*FGFR2*	NM_000141.5	Chr10:121517378C>A	c.1025G>T	p.Cys342Phe	CM960648	M, SA	I
25	P28	F	*FGFR2*	NM_000141.5	Chr10:121517377G>C	c.1026C>G	p.Cys342Trp	CM950468	N/A	N/A
26	P14	M	*FGFR2*	NM_000141.5	Chr10:121517342G>C	c.1061C>G	p.Ser354Cys	CM940784	CB, SA	S
27	P54	F	*FGFR2*	NM_000141.5	Chr10:121496701T>C	c.1694A>G	p.Glu565Gly	CM020141	CB, LB, M, SA	S
28	P3	F	*FGFR3*	NM_000142.5	Chr4:1801844C>G	c.749C>G	p.Pro250Arg	CM960655	CU	I
29	P7	F	*FGFR3*	NM_000142.5	Chr4:1801844C>G	c.749C>G	p.Pro250Arg	CM960655	CB	I
30	P11	F	*FGFR3*	NM_000142.5	Chr4:1801844C>G	c.749C>G	p.Pro250Arg	CM960655	CU, SA	I
31	P15	F	*FGFR3*	NM_000142.5	Chr4:1801844C>G	c.749C>G	p.Pro250Arg	CM960655	CU	I
32	P36	F	*FGFR3*	NM_000142.5	Chr4:1801844C>G	c.749C>G	p.Pro250Arg	CM960655	N/A	N/A
33	P37	F	*FGFR3*	NM_000142.5	Chr4:1801844C>G	c.749C>G	p.Pro250Arg	CM960655	N/A	N/A
34	P38	F	*FGFR3*	NM_000142.5	Chr4:1801844C>G	c.749C>G	p.Pro250Arg	CM960655	N/A	N/A
35	P39	F	*FGFR3*	NM_000142.5	Chr4:1801844C>G	c.749C>G	p.Pro250Arg	CM960655	N/A	N/A
36	P42	M	*FGFR3*	NM_000142.5	Chr4:1801844C>G	c.749C>G	p.Pro250Arg	CM960655	CB, SA	S
37	P45	F	*FGFR3*	NM_000142.5	Chr4:1801844C>G	c.749C>G	p.Pro250Arg	CM960655	CB	I
38	P46	M	*FGFR3*	NM_000142.5	Chr4:1801844C>G	c.749C>G	p.Pro250Arg	CM960655	CB, L	I
39	P135	F	*FGFR3*	NM_000142.5	Chr4:1806581C>T	c.2066C>T	p.Thr689Met	-	CU	I
40	P137	M	*KMT2A*	NM_001197104.2	Chr11:118436605_118436675del	c.93_163del	p.Arg32Leufs*91	-	LU, M, SA	S
41	P114	F	*KMT2D*	NM_003482.4	Chr12:49030893_49030901del	c.13663_13671del	p.Leu4555_Gln4557del	-	SA	S
42	P63	M	*MN1*	NM_002430.3	Chr22:28146983C>T	c.3883C>T	p.Arg1295	CM162266	M, SA	S
43	P58	M	*NSD1*	NM_022455.5	Chr5:177211351_177211352del	c.2954_2955del	p.Ser985Cysfs*25	CD054393	M	S
44	P99	F	*NSD1*	NM_022455.5	Chr5:177269630C>T	c.5332C>T	p.Arg1778*	CM030076	SA	S
45	P62	F	*RECQL4*	NM_004260.4	Chr8:144517096G>A	c.308C>T	p.Pro103Leu	CM033805	CB, M, SA	S
					Chr8:144512318C>T	c.3062G>A	p.Arg1021Gln	CM033810		
46	P119	M	*TCF12*	NM_207,037.2	Chr15:57166432T>C	c.356T>C	p.Leu119Pro	-	CB, LB, M, SA	I
47	P106	F	*TCF12*	NM_207,037.2	Chr15:57231251del	c.679del	p.Met227Cysfs*18	-	CU	I
48	P64	F	*TCF12*	NM_207,037.2	Chr15:57232818C>G	c.932C>G	p.Ser311*	-	CB	S
49	P70	F	*TCF12*	NM_207,037.2	Chr15:57282482_57282483ins	c.2015_2016ins	p.Arg672Serfs*2	-	CB, LB, M, SA	I
50	P44	F	*TWIST1*	NM_000474.4	Chr7:19117225	c.97A>T	p.Lys33*	-	N/A	N/A
51	P4	F	*TWIST1*	NM_000474.4	Chr7:19117170C>A	c.152G>T	p.Gly51Val	-	CB, LB, SA	S
53	P40	M	*TWIST1*	NM_000474.4	Chr7:19117063_19117065dup	c.257_259dup	p.Gly86dup	-	M, SA	S
53	P41	F	*TWIST1*	NM_000474.4	Chr7:19117063_19117065dup	c.257_259dup	p.Gly86dup	-	CB, LB, SA, M	S
54	P128	M	*TWIST1*	NM_000474.4	Chr7:19117063_19117065dup	c.257_259dup	p.Gly86dup	-	CU	S
55	P8	F	*TWIST1*	NM_000474.4	Chr7:19117043_19117044ins	c.279_280ins	p.Ser94Glyfs*146	-	C, L, SA	I
56	P9	M	*TWIST1*	NM_000474.4	Chr7:19117043_19117044ins	c.279_280ins	p.Ser94Glyfs*146	-	CB, M	I
57	P17	F	*TWIST1*	NM_000474.4	Chr7:19116973C>A	c.349G>T	p.Glu117*	-	N/A	N/A
58	P18	F	*TWIST1*	NM_000474.4	Chr7:19116973C>A	c.349G>T	p.Glu117*	-	N/A	N/A
59	P13	M	*TWIST1*	NM_000474.4	Chr7:19116954G>T	c.368C>A	p.Ser123*	CM970033	CU	S
60	P19	M	*TWIST1*	NM_000474.4	Chr7:19116946C>A	c.376G>T	p.Glu126*	CM970034	CB, SA	I
61	P20	M	*TWIST1*	NM_000474.4	Chr7:19116946C>A	c.376G>T	p.Glu126*	CM970034	N/A	I
62	P43	F	*TWIST1*	NM_000474.4	Chr7:19116906_19116927dup	c.395_416dup	p.Ser140Glufs*105	—	N/A	N/A
63	P1	F	*TWIST1*	NM_000474.4	Chr7:19116867G>A	c.455C>T	p.Ala152Val	CM980027	Acrocephaly	I
64	P2	F	*TWIST1*	NM_000474.4	Chr7:19116867G>A	c.455C>T	p.Ala152Val	CM980027	CB	I
65	P16	M	*TWIST1*	NM_000474.4	Chr7:19116774A>C	c.548T>G	p.Leu183Arg	—	CU	I
66	P129	F	*ZIC1*	NM_003412.4	Chr3:147413379C>A	c.1172C>A	p.Ser391*	—	C, LU, M, SA	S
67	P130	M	*ZIC1*	NM_003412.4	Chr3:147131204T>C	c.1210T>C	p.Ser404Pro	—	CU, SA	I

### Tier 2

We have detected four CNVs in four individuals using CMA, i.e., three duplications in locus 1q22-q23.1, locus 2p21 encompassing solely the *SIX2* gene, locus 17p13.3, and one deletion in locus 5q35.3, which included exons 18–21 in the *NSD1* gene (Table 3; Supplementary Table S5) (Sowińska-Seidler et al., 2018). Targeted NGS of a custom gene panel allowed us to establish the molecular diagnosis in the subsequent 14 sporadic patients (15 variants) (Table 2; Supplementary Table S6). We have found 15 following heterozygous variants, from which 9 were not reported in HGMD–c.578_581del p.Thr193Argfs*137 in the *ALX3* gene (variant of unknown significance, VUS), c.491A>G p. Asp164Gly in the *EFTUD2* gene, c.394C>T p.Arg132* in the *ERF* gene (linked to Craniosynostosis 4), c.868T>G p.Trp290Gly (HGMD no: CM1313533), c.1025G>A p.Cys342Tyr (HGMD no: CM940779), c.1694A>G p.Glu565Gly (HGMD no: CM020141) in the *FGFR2* gene (HGMD no: CM020141), and c.2066C>T p.Thr689Met in the *FGFR3* gene, c.356T>C p.Leu119Pro, c.679del p.Met227Cysfs*18, c.932C>G p.Ser311*, c.2015_2016ins p.Arg672Serfs*2 in the *TCF12* gene (linked to Craniosynostosis 3), c.1172C>A p.Ser391*, c.1210T>C p.Ser404Pro (VUS) in the *ZIC1* gene (linked to Craniosynostosis 6) and two alterations in compound heterozygosity c.308C>T p.Pro103Leu (HGMD no: CM033805), and c.3062G>A p.Arg1021Gln (HGMD no: CM033810) located within the *RECQL4* gene (linked to Rothmund-Thomson, Baller-Gerold, and RAPADILINO syndromes).

**TABLE 3:** The list of *de novo* aberrations and copy number variations (CNVs) found in the cohort of 166 patients affected by syndromic craniosynostosis. ISCN, International System for Human Cytogenetic Nomenclature; N/A, not applicable. P140 was diagnosed with Sotos syndrome, P142 with 17p13.3 microduplication syndrome class I.

#	Patient ID	Sex	Locus	ISCN	Size	Affected suture(s)	Candidate Gene	Additional Phenotype
**1**	P138*	M	1q22-q23.1	arr[GRCh38] 1q22-q23.1(chr1:155961428–157217426)x3	1.3 Mb	Metopic, lambdoid unilateral	*BGLAP*, *LMNA*	Global developmental delay, hypotonia, facial dysmorphism, low-set, posteriorly rotated ears
**2**	P139	M	2p21	arr[GRCh38]2p21(chr2:44990857–45008348)x3	17.5 kb	Metopic, sagittal	*SIX2*	Hyperactivity, ptosis, angioma of the right eye socket, broad nasal bridge, hypertelorism, microcephaly, mild intellectual disability, delayed myelinization, right cryptorchidism, hydronephrosis, recurrent respiratory infections, one cafe au lait spot on the right thigh
**3**	P140	M	5q35.3	arr[GRCh38]5q35.3(chr5:177277901–177283748)x1	5.8 kb	Sagittal, lambdoid bilateral	*NSD1*	Macrocephaly, micrognathia, retrognathia, high arched palate, cleft palate, bilateral hearing loss, recurrent otitis media, anaplastic ears lobes, umbilical hernia, macrosomia
**4**	P141**	F	7q32.3-q35	arr[GRCh38]7q32.3-q35(chr7:131837067–144607071)x1	12.8 Mb	Coronal bilateral, sagittal	*BRAF*	Facial dysmorphism: proptosis, hypertelorism, down-slanted palpebral fissures, broad nasal bridge, and bulbous nasal tip, intellectual disability, delayed psychomotor development, delayed speech, increased intracranial pressure
**5**	P142	M	17p13.3	arr[GRCh38]17p13.3(chr17:847,955–1641,601)x3	793.6 kb	Metopic	*YWHAE*, *CRK*	Facial asymmetry, short frenum, heart defect (PFO), cryptorchidism, hypotonia, psychomotor delay
**6**	P143	F	18q21.32-q23	arr[GRCh38]18q21.32-q23(chr18:620405559–80247,644)x1	21.8 Mb	Coronal unilateral	N/A	Global developmental delay, speech delay, heart defect (FoA), hearing loss

Note: this data are partially retrospective studies as CNVs, detected in P138 and P141 have been already published by our team *[20]; **[21] ^#^Exome-sequencing analysis.

### Tier 3

Finally, applying ES, we revealed seven heterozygous variants in the subsequent seven patients–c.791C>A p.Ser264* in the *ARID1A* gene (linked to Coffin-Siris type 2 syndrome), c.93_163del p.Arg32Leufs*91 in the *KMT2A* gene (linked to Wiedemann-Steiner syndrome), c.13663_13671del p.Leu4555_Gln4557del in the *KMT2D* gene (linked to Kabuki type 1 syndrome), c.3883C>T p.Arg1295* in the *MN1* gene (linked to MN1 C-terminal truncation syndrome; MCTT syndrome, and CEBALID syndrome), c.2954_2955del p.Ser985Cysfs*25, and c.5332C>T p.Arg1778* in the *NSD1* gene (linked to Sotos type 1 syndrome), and c.913A>G p.Arg305Gly in *FAM111A* (linked to Gracile bone dysplasia, and Kenny-Caffey syndrome type 2) (Table 2; Supplementary Table S6). Those variants were not reported in the medical literature, except for mutations detected in the *NSD1* gene–p.Ser985Cysfs*25 (HGMD no: CD054393), and p.Arg1778* (HGMD no: CM030076).

## Discussion

Craniosynostosis represents a highly heterogeneous medical condition whose etiology has not been yet fully elucidated. The results obtained from cohorts screened worldwide showed that the molecular background could be indicated in merely 21%, to propitiously 62% of patients (Roscioli et al., 2013; Paumard-Hernandez et al., 2015; Timberlake et al., 2016; Lee et al., 2018; Topa et al., 2020). Positive genetic testing is mainly achieved among subgroups with syndromic CS. The reported germline mutations are usually classified as point mutations, however, chromosomal aberrations, copy number variations, minor exonic deletions/duplication, or biallelic inheritance were also reported in the medical literature (Timberlake et al., 2016; Goos and Mathijssen, 2019; Yilmaz et al., 2019).

**FIGURE 1:**
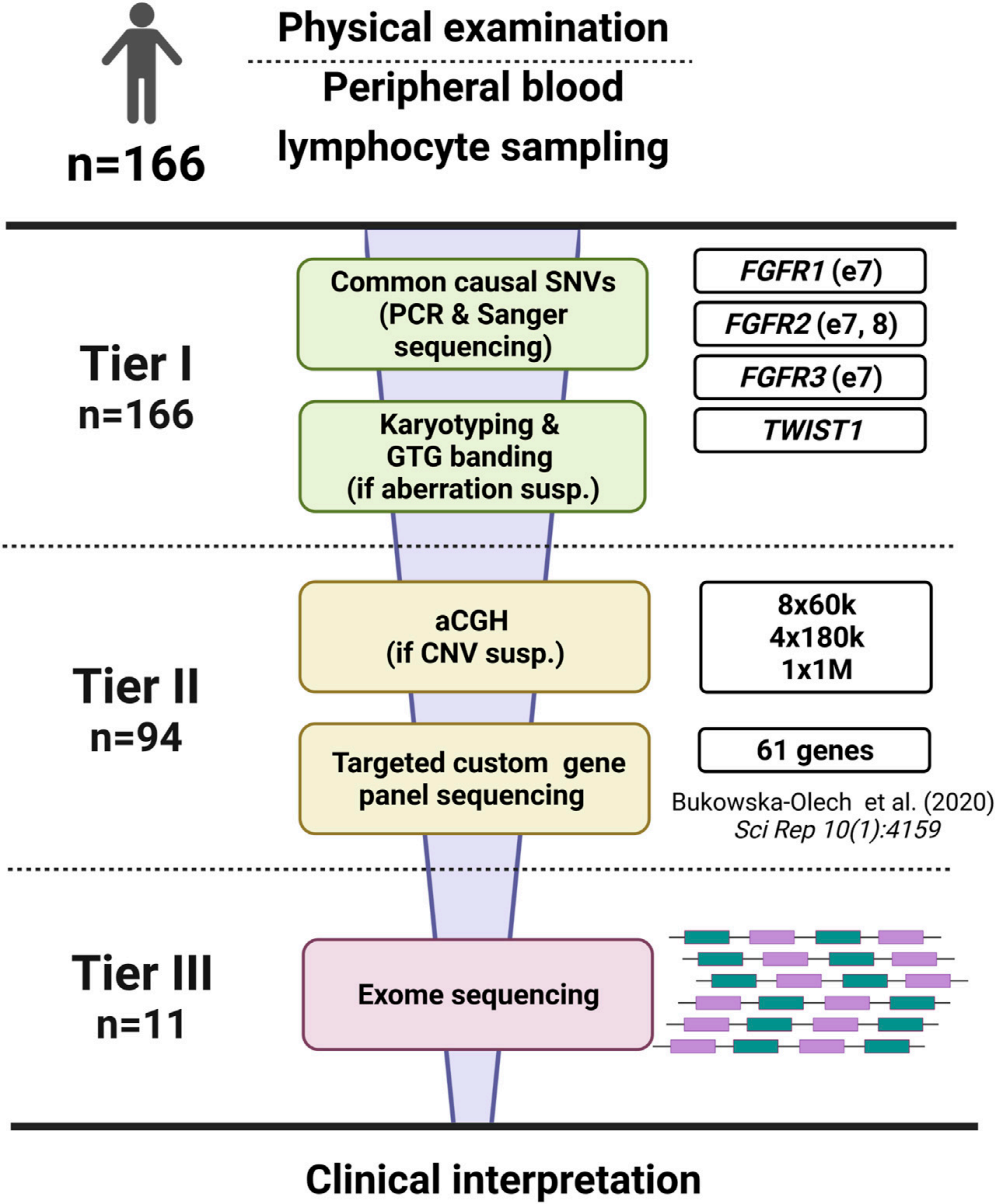
The scheme of the diagnostic algorithm applied in our study regarding 166 patients affected with craniosynostosis. The algorithm was divided into three tiers.

In this study, we have reported 166 individuals affected with different forms of CS in whom we had applied a three-step diagnostic algorithm (Tier 1–3) (Figure 1). To our best knowledge, this is the first large CS patients’ screening in which multi-leveled methods, including chromosomal aberrations and CNVs detection, were applied. The proposed approach allowed us to identify an exact genetic cause in around 43% of all CS patients. Since our multi-leveled molecular diagnostic strategy of CS patients is unique and previously unreported, we were unable to directly compare all the results obtained here with previous similar research studies. This is because all reports that have been submitted so far, were aimed at identifying only point mutations *via* targeted Sanger sequencing, targeted gene panel NGS or ES. Similar to other researchers analyzing these mutations type occurrence, we have shown that causative variants in the *FGFR1*, *FGFR2*, *FGFR3*, *TWIST1*, and *TCF12* genes account for most common cause of CS (Roscioli et al., 2013; Paumard-Hernandez et al., 2015; Lee et al., 2018; Topa et al., 2020).

Notably, *FGFR1*, *FGFR2*, *FGFR3* variants mainly occur in hot-spot positions and, along with *TWIST1* gene mutations, were analyzed in Tier 1. Undeniably, this step was crucial in the CS testing algorithm and cost-effective compared to other genetic methods (73% of all diagnoses;–χ^2^ (1;71) = 14.43; *p* < 0.001). On the other hand, four novel variants in the *TCF12* gene and additional *FGFR2*, *FGFR3* alterations (other than hot-spots) were found using custom gene panel NGS (Table 2). No other gene included in the applied custom gene panel housed more than one pathogenic variant. Interestingly, based on the medical literature, the *EFNB1* gene is usually reported as the sixth most commonly affected gene in CS (Paumard-Hernandez et al., 2015; Miller et al., 2017; Lee et al., 2018). However, we postulate that a very characteristic disease, i.e., craniofrontonasal dysplasia (CFND) resulting from pathogenic variants of the *EFNB1*, should be considered a standalone genetic disorder in which features other than CS guide the proper diagnosis (Bukowska-Olech et al., 2021). Moreover, CFND represents a classic viscerocranium defect, whereas CS is a neurocranium abnormality. Hence, we have excluded all individuals suggestive of CFND and consequently have not reported *EFNB1* mutations in this study.

Next, we have revealed that six patients with syndromic CS carried pathogenic variants in genes involved in epigenetic regulations such as *ARID1A* (P134), *KMT2A* (P137), *KMT2D* (P114), *NSD1* (P58, P99, P166) (Table 2), resulting in Coffin-Siris syndrome type 2, Wiedemann-Steiner syndrome, Kabuki syndrome type 1, and Sotos syndrome type 1, respectively. Such Mendelian disorders, i.e., those resulting from disruptions of epigenetic processes, were termed chromatinopathies. They are all characterized by intellectual disability, immune deficiencies, or skeletal anomalies. However, CS was rarely described among them (Zollino et al., 2017). Occasionally, CS has been reported only in Kabuki syndrome and Sotos syndrome thus far (Tatton-Brown et al., 2005; Martínez-Lage et al., 2010; Topa et al., 2017). The second group of Mendelian diseases in which CS has been noted are RASopathies resulting from the dysregulation of RAS/MAPK pathway. Retrospectively, we have described here one patient carrying CNVs in which *BRAF* gene deletion occurred (P141) (Bukowska-Olech et al., 2020b). Similar to our finding, other researchers have also highlighted the co-occurrence of both CS and RASopathy, resulting from mutations in other components of RAS/MAPK pathway (Kratz et al., 2009; Takenouchi et al., 2014; Ueda et al., 2017).

Custom genes panel allowed us to detect novel pathogenic variants–p.Arg132* in the *ERF* gene (P129), and p.Ser391* in the *ZIC1* gene (P131), resulting in Craniosynostosis 4 and Craniosynostosis 6, respectively (Twigg et al., 2013, 2015). Both *ERF* and *ZIC1* are newly recognized CS-related genes, however, only a few cases carrying variants in those two have been reported (Twigg et al., 2015; Miller et al., 2017; Glass et al., 2019). In addition, we have evaluated one variant in the *ZIC1* gene as VUS p.Ser404Pro (P130) since it was present in the patient’s healthy father. However, this alteration was absent from the gnomAD v3.1.2 database (accession date: 3 December 2021). Finally, ES revealed two additional alterations in individuals with syndromic CS–p.Arg1295* in the *MN1* (P63), which was recently discovered, and subsequently linked to CS, and p.Arg305Gly in *FAM111A* (P136), resulting in Kenny-Caffey syndrome type 2, in which CS represents an unseen clinical feature (Table 2) (Mak et al., 2020).

Importantly, we have noted an apparent gap regarding screening for chromosomal aberrations or CNVs among CS patients (Lattanzi et al., 2012; Poot, 2019). To our knowledge, no major CS groups were analyzed *via* karyotyping or CMA, therefore most research data describing microscopic chromosomal changes or submicroscopic CNVs causative for CS were published as single case reports (Villa et al., 2007; Marques et al., 2015). Besides, only a few chromosomal aberrations and CNVs known thus far represent recurrent changes underlying CS (e.g., deletions in 7p21, 9p22-p24, and 11q23-q24 or duplication in 5q33.3), however none of them was present in our cohort (Reardon et al., 1993; Shiihara et al., 2004; Poot, 2019). All genomic losses or gains reported in this research were not commonly associated with CS (Budisteanu et al., 2010; Dilzell et al., 2015). Hence, we could not recommend additional loci to be screened regarding the cohort of syndromic CS, especially those associated with intellectual disability. Here, karyotyping followed by GTG banding allowed us to detect two intrachromosomal deletions 7q32.3-q35, and 18q21.32-q23, both resized to chr7:131837067-144607071, and chr18:620405559-80247644, respectively. Next, using CMA, we have detected four CNVs including three duplications–1q22-q23.1 (chr1:155961428-157217426), 2p21 (chr2:44990857-45008348), 17p13.3 (chr17:847955-1641601), and one deletion 5q35.3 (chr5:177277901-177283748) from which the largest encompassed 1.3 Mb, whereas the smallest 5.8 kb (Sowińska-Seidler et al., 2018; Bukowska-Olech et al., 2020a). The detailed mutations description following the International System for Human Cytogenomic Nomenclature (ISCN) was listed in Table 3; for a list of genomic mutations’ content, see Supplementary Table S5. Notably, in most chromosomal aberrations or CNVs identified here, CS occurred as an additional phenotype. The above findings suggest that karyotyping and CMA cannot be replaced by targeted chromosomal testing when applied in a cohort of syndromic CS associated with an intellectual disability or developmental delay.

Regarding the results presented in this study, we would like to point to the possible underestimated co-occurrence of CS and intellectual disability. Our clinical experience suggests that CS may be overlooked when intellectual disability constitutes a primary clinical complaint. Hence, we recommend calvarial sutures’ evaluation in patients with intellectual disability. On the other hand, in any case of already detected syndromic CS and intellectual disability, the possible occurrence of clinical features suggestive for either chromatinopathies or RASopathies should also be considered (Kratz et al., 2009; Cao et al., 2017; Zollino et al., 2017; Davis et al., 2019).

**FIGURE 2:**
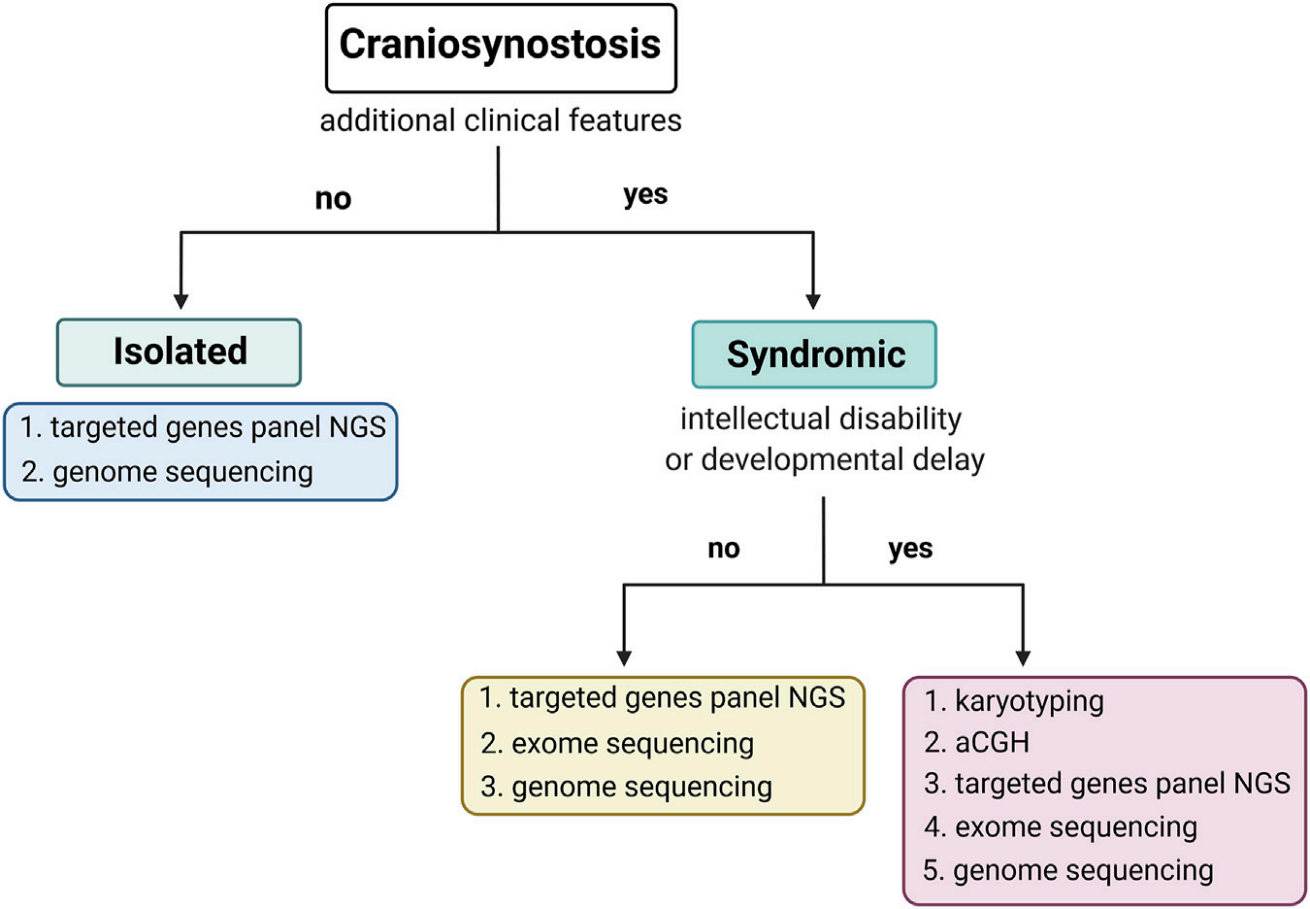
The scheme of the proposed diagnostic algorithm was prepared based on results obtained from the current study.

Undeniably, the molecular diagnosis of CS should distinguish its isolated or syndromic form, which presence determines the subsequent diagnostic steps. In addition, syndromic CS should be classified as a disorder associated with intellectual disability or a disorder without intellectual disability (Figure 2). Targeted PCR and Sanger sequencing of *FGFR1*, *FGFR2*, *FGFR3*, *TWIST1*, and *TCF12* genes resulted in the highest diagnostic rate in our cohort of craniosynostosis patients strongly recommend analysing those genes first (isolated CS and syndromic CS without intellectual disability). Because of many advantages of NGS-based methods, including mosaicism detection, screening those genes using targeted genes panel *via* NGS would be optimal. However, based on our results, we suggest applying a custom genes panel limited to the fewer genes, such as *FGFR1*, *FGFR2*, *FGFR3*, *TWIST1*, *TCF12*, *ERF*, *ZIC1, RECQL4,* and *NSD1* (Siitonen et al., 2009; Sharma et al., 2013; Twigg et al., 2013, Twigg et al., 2015; Twigg and Wilkie, 2015; Miller et al., 2017; Kutkowska-Kaźmierczak et al., 2018; Lee et al., 2018; Bukowska-Olech et al., 2020c; Topa et al., 2020)*.* In the case of syndromic CS and intellectual disability, the genetic investigation should start from chromosomal aberrations or CNVs detection. Lastly, when ES data bioinformatic analysis is performed, genes associated with RASopathies and chromatinopathies should be considered.

The heterogeneity of CS is enormous, resulting from either anatomical variability of the disorder, in which different types and number of sutures can be affected, or epidemiological aspects, including isolated presentation of CS or occurrence of various accompanying symptoms (Kutkowska-Kazmierczak et al., 2018). It has also been shown that CS may be detected in at least 180 different syndromes, which often are rare and in which CS is not a pathognomic feature. Besides, some researchers have also documented or postulated the association between CS and two-locus inheritance. Consequently, molecular causes of the disease seem to be complex in some CS individuals and, as presented in this study, the pathogenic variant or affected gene may be restricted to only one individual (Sharma et al., 2013; Flaherty et al., 2016; Timberlake et al., 2016, Timberlake et al., 2018; Wilkie et al., 2007; Timberlake and Persing, 2018). However, in many CS patients, including our cohort, genetic causes of observed phenotypes remain unrevealed. One may suspect the presence of deep-intronic and regulatory variants, polygenic inheritance, or even epigenetic influences. Another explanation may be the technical limitations of currently available diagnostic methods. It has been shown, for example, that the application of long-read sequencing in NGS-based methods may clarify the genetic background in many unresolved cases (Fujimoto et al., 2021; Hiatt et al., 2021; Miller et al., 2021; Rastegar and Yasui, 2021). Considering the above, the next steps that we should consider to implement for diagnosis of our unsolved cases include whole-genome sequencing, RNA-seq, whole-genome bisulfite sequencing, or long-read ES.

To conclude, our research may constitute a significant source of epidemiological information as we have presented precise phenotypic and genetic data derived from 166 consecutive CS patients of Caucasian origin. We yielded a 43% diagnostic success rate using the presented approach, highlighting the high occurrence of pathogenic variants within “classic” CS genes, i.e., *FGFR1*, *FGFR2*, *FGFR3*, *TWIST1*, and *TCF12*. Moreover, we have critically summarized the applied diagnostic methods (Figure 1) and proposed the optimized, cost-effective diagnostic algorithm, which may be helpful in a daily diagnostic routine of various CS’ types (Figure 2).

### Web Resources

Database of Genomic Variants (http://dgv.tcag.ca/dgv/app/home).

DECIPHER (https://www.deciphergenomics.org/browser).

Human Gene Mutation database (http://www.hgmd.cf.ac.uk/ac/index.php).

Human Phenotype Ontology (https://hpo.jax.org/app/).

Online Mendelian Inheritance in Man (https://www.omim.org).

Primer3 tool (http://bioinfo.ut.ee/primer3-0.4.0/).

UCSC Genome Browser (https://genome.ucsc.edu/index.html).

Varsome (https://varsome.com/).

## Data Availability

The datasets generated during and/or analyzed during the current study are available from the corresponding authors on reasonable request.

## References

[B1] Baer M. J. (1954). Patterns of Growth of the Skull as Revealed by Vital Staining. Hum. Biol. 26, 80–126. 13191803

[B2] Budisteanu M. Arghir A. Chirieac S. M. Tutulan-Cunita A. Lungeanu A. (2010). 18q Deletion Syndrome - A Case Report. Maedica (Bucur) 5, 135–138. 21977138 PMC3150013

[B3] Bukowska‐Olech E. Dmitrzak‐Węglarz M. Larysz D. Wojciechowicz B. Simon D. Walczak‐Sztulpa J. (2020a). Compound Craniosynostosis, Intellectual Disability, and Noonan‐Like Facial Dysmorphism Associated with 7q32.3‐q35 Deletion. Birth Defects Res. 112, 740–748. 10.1002/bdr2.1744 32529787

[B4] Bukowska‐Olech E. Dmitrzak‐Węglarz M. Larysz D. Wojciechowicz B. Simon D. Walczak‐Sztulpa J. (2020b). Compound Craniosynostosis, Intellectual Disability, and Noonan‐Like Facial Dysmorphism Associated with 7q32.3‐q35 Deletion. Birth Defects Res. 112, 740–748. 10.1002/bdr2.1744 32529787

[B5] Bukowska-Olech E. Gawliński P. Jakubiuk-Tomaszuk A. Jędrzejowska M. Obersztyn E. Piechota M. (2021). Clinical and Molecular Characterization of Craniofrontonasal Syndrome: New Symptoms and Novel Pathogenic Variants in the EFNB1 Gene. Orphanet J. Rare Dis. 16, 286. 10.1186/s13023-021-01914-1 34174922 PMC8236199

[B6] Bukowska-Olech E. Popiel D. Koczyk G. Sowińska-Seidler A. Socha M. Wojciechowicz B. (2020c). Adapting SureSelect Enrichment Protocol to the Ion Torrent S5 Platform in Molecular Diagnostics of Craniosynostosis. Sci. Rep. 10, 4159. 10.1038/s41598-020-61048-5 32139749 PMC7058001

[B7] Cao H. Alrejaye N. Klein O. D. Goodwin A. F. Oberoi S. (2017). A Review of Craniofacial and Dental Findings of the RASopathies. Orthod. Craniofac. Res. 20 (Suppl. 1), 32–38. 10.1111/ocr.12144 28643916 PMC5942188

[B8] Chieffo D. Tamburrini G. Massimi L. Di Giovanni S. Giansanti C. Caldarelli M. (2010). Long-Term Neuropsychological Development in Single-Suture Craniosynostosis Treated Early. Ped 5, 232–237. 10.3171/2009.10.PEDS09231 20192638

[B9] Davis A. A. Zuccoli G. Haredy M. M. Losee J. Pollack I. F. Madan-Khetarpal S. (2019). RASopathy in Patients with Isolated Sagittal Synostosis. Glob. Pediatr. Health 6, 2333794X1984677–2333794X19846774. 10.1177/2333794X19846774 PMC6540476 31192281

[B10] Dilzell K. Darcy D. Sum J. Wallerstein R. (20152015). Deletion of 7q33-Q35 in a Patient with Intellectual Disability and Dysmorphic Features: Further Characterization of 7q Interstitial Deletion Syndrome. Case Rep. Genet. 2015, 1–5. 10.1155/2015/131852 PMC4433680 26064708

[B11] Fujimoto A. Wong J. H. Yoshii Y. Akiyama S. Tanaka A. Yagi H. (2021). Whole-Genome Sequencing with Long Reads Reveals Complex Structure and Origin of Structural Variation in Human Genetic Variations and Somatic Mutations in Cancer. Genome Med. 13, 65. 10.1186/s13073-021-00883-1 33910608 PMC8082928

[B12] Glass G. E. O'Hara J. Canham N. Cilliers D. Dunaway D. Fenwick A. L. (2019). ERF-Related Craniosynostosis: The Phenotypic and Developmental Profile of a New Craniosynostosis Syndrome. Am. J. Med. Genet. 179, 615–627. 10.1002/ajmg.a.61073 30758909 PMC6491982

[B13] Goos J. A. C. Mathijssen I. M. J. (2019). Genetic Causes of Craniosynostosis: An Update. Mol. Syndromol 10, 6–23. 10.1159/000492266 30976276 PMC6422124

[B14] Gupta P. C. Foster J. Crowe S. Papay F. A. Luciano M. Traboulsi E. I. (2003). Ophthalmologic Findings in Patients with Nonsyndromic Plagiocephaly. J. Craniofac. Surg. 14, 529–532. 10.1097/00001665-200307000-00026 12867869

[B15] Hiatt S. M. Lawlor J. M. J. Handley L. H. Ramaker R. C. Rogers B. B. Partridge E. C. (2021). Long-Read Genome Sequencing for the Molecular Diagnosis of Neurodevelopmental Disorders. Hum. Genet. Genomics Adv. 2, 100023. 10.1016/j.xhgg.2021.100023 PMC8087252 33937879

[B16] Johnson D. Wilkie A. O. M. (2011). Craniosynostosis. Eur. J. Hum. Genet. 19, 369–376. 10.1038/ejhg.2010.235 21248745 PMC3060331

[B17] Kratz C. P. Zampino G. Kriek M. Kant S. G. Leoni C. Pantaleoni F. (2009). Craniosynostosis in Patients with Noonan Syndrome Caused by Germline KRAS Mutations. Am. J. Med. Genet. 149A, 1036–1040. 10.1002/ajmg.a.32786 19396835

[B18] Kutkowska-Kaźmierczak A. Gos M. Obersztyn E. (2018). Craniosynostosis as a Clinical and Diagnostic Problem: Molecular Pathology and Genetic Counseling. J. Appl. Genet. 59, 133–147. 10.1007/s13353-017-0423-4 29392564

[B19] Lattanzi W. Barba M. Di Pietro L. Boyadjiev S. A. (2017). Genetic Advances in Craniosynostosis. Am. J. Med. Genet. 173, 1406–1429. 10.1002/ajmg.a.38159 28160402 PMC5397362

[B20] Lattanzi W. Bukvic N. Barba M. Tamburrini G. Bernardini C. Michetti F. (2012). Genetic Basis of Single-Suture Synostoses: Genes, Chromosomes and Clinical Implications. Childs Nerv Syst. 28, 1301–1310. 10.1007/s00381-012-1781-1 22872241

[B21] Lee E. Le T. Zhu Y. Elakis G. Turner A. Lo W. (2018). A Craniosynostosis Massively Parallel Sequencing Panel Study in 309 Australian and New Zealand Patients: Findings and Recommendations. Genet. Med. 20, 1061–1068. 10.1038/gim.2017.214 29215649

[B22] Mak C. C. Y. Doherty D. Lin A. E. Vegas N. Cho M. T. Viot G. (2020). MN1 C-Terminal Truncation Syndrome Is a Novel Neurodevelopmental and Craniofacial Disorder with Partial Rhombencephalosynapsis. Brain. 143, 55–68. 10.1093/brain/awz379 31834374 PMC7962909

[B23] Marques F. Heredia R. de Oliveira C. Cardoso M. T. Mazzeu J. Pogue R. (2015). Partial Trisomy 17q and Partial Monosomy 20q in a Boy with Craniosynostosis. Am. J. Med. Genet. 167, 412–416. 10.1002/ajmg.a.36844 25424318

[B24] Martínez-Lage J. F. Felipe-Murcia M. Navarro E. G. Almagro M.-J. López-Guerrero A. L. Pérez-Espejo M. A. (2010). Craniosynostosis in Kabuki Syndrome. Ped 6, 198–201. 10.3171/2010.5.PEDS09286 20672944

[B25] Mathijssen I. M. J. Arnaud E. (2007). Benchmarking for Craniosynostosis. J. Craniofac. Surg. 18, 436–442. 10.1097/scs.0b013e31802d4c6c 17414298

[B26] Miller D. E. Sulovari A. Wang T. Loucks H. Hoekzema K. Munson K. M. (2021). Targeted Long-Read Sequencing Identifies Missing Disease-Causing Variation. Am. J. Hum. Genet. 108, 1436–1449. 10.1016/j.ajhg.2021.06.006 34216551 PMC8387463

[B27] Miller K. A. Twigg S. R. F. McGowan S. J. Phipps J. M. Fenwick A. L. Johnson D. (2017). Diagnostic Value of Exome and Whole Genome Sequencing in Craniosynostosis. J. Med. Genet. 54, 260–268. 10.1136/jmedgenet-2016-104215 27884935 PMC5366069

[B28] Morriss-Kay G. M. Wilkie A. O. M. (2005). Growth of the Normal Skull Vault and its Alteration in Craniosynostosis: Insights From Human Genetics and Experimental Studies. J. Anat. 207, 637–653. 10.1111/j.1469-7580.2005.00475.x 16313397 PMC1571561

[B29] Opperman L. A. (2000). Cranial Sutures as Intramembranous Bone Growth Sites. Dev. Dyn. 219, 472–485. 10.1002/1097-0177(2000)9999:9999<::aid-dvdy1073>3.0.co;2-f 11084647

[B30] Paumard-Hernández B. Berges-Soria J. Barroso E. Rivera-Pedroza C. I. Pérez-Carrizosa V. Benito-Sanz S. (2015). Expanding the Mutation Spectrum in 182 Spanish Probands with Craniosynostosis: Identification and Characterization of Novel TCF12 Variants. Eur. J. Hum. Genet. 23, 907–914. 10.1038/ejhg.2014.205 25271085 PMC4463497

[B31] Poot M. (2019). Structural Genome Variations Related to Craniosynostosis. Mol. Syndromol. 10, 24–39. 10.1159/000490480 30976277 PMC6422139

[B32] Rastegar M. Yasui D. H. (2021). Editorial: Epigenetic Mechanisms and Their Involvement in Rare Diseases. Front. Genet. 12, 755076. 10.3389/fgene.2021.755076 34539761 PMC8440956

[B33] Reardon W. McManus S. P. Summers D. Winter R. M. (1993). Cytogenetic Evidence that the Saethre-Chotzen Gene Maps to 7p21.2. Am. J. Med. Genet. 47, 633–636. 10.1002/ajmg.1320470510 8266988

[B34] Renier D. Sainte-Rose C. Marchac D. Hirsch J.-F. (1982). Intracranial Pressure in Craniostenosis. J. Neurosurg. 57, 370–377. 10.3171/jns.1982.57.3.0370 7097333

[B35] Rice D. P. (2008). Developmental Anatomy of Craniofacial Sutures. Front. Oral Biol. 12, 1–21. 10.1159/000115028 18391492

[B36] Richards S. Aziz N. Bale S. Bick D. Das S. Gastier-Foster J. (2015). Standards and Guidelines for the Interpretation of Sequence Variants: A Joint Consensus Recommendation of the American College of Medical Genetics and Genomics and the Association for Molecular Pathology. Genet. Med. 17, 405–424. 10.1038/gim.2015.30 25741868 PMC4544753

[B37] Roscioli T. Elakis G. Cox T. C. Moon D. J. Venselaar H. Turner A. M. (2013). Genotype and Clinical Care Correlations in Craniosynostosis: Findings from a Cohort of 630 Australian and New Zealand Patients. Am. J. Med. Genet. 163, 259–270. 10.1002/ajmg.c.31378 24127277

[B38] Sharma V. P. Fenwick A. L. Fenwick A. L. Brockop M. S. McGowan S. J. Goos J. A. C. (2013). Mutations in TCF12, Encoding a Basic Helix-Loop-Helix Partner of TWIST1, Are a Frequent Cause of Coronal Craniosynostosis. Nat. Genet. 45, 304–307. 10.1038/ng.2531 23354436 PMC3647333

[B39] Shiihara T. Kato M. Kimura T. Hayasaka K. Yamamori S. Ogata T. (2004). Craniosynostosis with Extra Copy of MSX2 in a Patient with Partial 5q-Trisomy. Am. J. Med. Genet. 128A, 214–216. 10.1002/ajmg.a.20552 15214020

[B40] Siitonen H. A. Sotkasiira J. Biervliet M. Benmansour A. Capri Y. Cormier-Daire V. (2009). The Mutation Spectrum in RECQL4 Diseases. Eur. J. Hum. Genet. 17, 151–158. 10.1038/ejhg.2008.154 18716613 PMC2986053

[B41] Sowińska-Seidler A. Olech E. M. Socha M. Larysz D. Jamsheer A. (2018). Novel 1q22-q23.1 Duplication in a Patient with Lambdoid and Metopic Craniosynostosis, Muscular Hypotonia, and Psychomotor Retardation. J. Appl. Genet. 59, 281–289. 10.1007/s13353-018-0447-4 29845577 PMC6060980

[B42] Takenouchi T. Sakamoto Y. Miwa T. Torii C. Kosaki R. Kishi K. (2014). Severe Craniosynostosis with Noonan Syndrome Phenotype Associated with *SHOC2* Mutation: Clinical Evidence of Crosslink Between FGFR and RAS Signaling Pathways. Am. J. Med. Genet. 164, 2869–2872. 10.1002/ajmg.a.36705 25123707

[B43] Tatton-Brown K. Douglas J. Coleman K. Baujat G. Cole T. R. P. Das S. (2005). Genotype-Phenotype Associations in Sotos Syndrome: An Analysis of 266 Individuals with NSD1 Aberrations. Am. J. Hum. Genet. 77, 193–204. 10.1086/432082 15942875 PMC1224542

[B44] Thompson D. N. P. Malcolm G. P. Jones B. M. Harkness W. J. Hayward R. D. (1995). Intracranial Pressure in Single-Suture Craniosynostosis. Pediatr. Neurosurg. 22, 235–240. 10.1159/000120907 7547454

[B45] Timberlake A. T. Choi J. Zaidi S. Lu Q. Nelson-Williams C. Brooks E. D. (2016). Two Locus Inheritance of Non-Syndromic Midline Craniosynostosis via Rare SMAD6 and Common BMP2 Alleles. eLife 5, e20125. 10.7554/eLife.20125 27606499 PMC5045293

[B46] Topa A. Rohlin A. Andersson M. K. Fehr A. Lovmar L. Stenman G. (2020). NGS Targeted Screening of 100 Scandinavian Patients with Coronal Synostosis. Am. J. Med. Genet. 182, 348–356. 10.1002/ajmg.a.61427 31837199

[B47] Topa A. Samuelsson L. Lovmar L. Stenman G. Kölby L. (2017). On the Significance of Craniosynostosis in a Case of Kabuki Syndrome with a Concomitant KMT2D Mutation and 3.2 Mbp De Novo 10q22.3q23.1 Deletion. Am. J. Med. Genet. 173, 2219–2225. 10.1002/ajmg.a.38296 28590022

[B48] Tubbs R. S. Elton S. Blount J. P. Oakes W. J. (2001). Preliminary Observations on the Association Between Simple Metopic Ridging in Children without Trigonocephaly and the Chiari I Malformation. Pediatr. Neurosurg. 35, 136–139. 10.1159/000050407 11641622

[B49] Twigg S. R. F. Forecki J. Goos J. A. C. Richardson I. C. A. Hoogeboom A. J. M. van den Ouweland A. M. W. (2015). Gain-of-Function Mutations in ZIC1 Are Associated with Coronal Craniosynostosis and Learning Disability. Am. J. Hum. Genet. 97, 378–388. 10.1016/j.ajhg.2015.07.007 26340333 PMC4564895

[B50] Twigg S. R. F. Vorgia E. McGowan S. J. Peraki I. Fenwick A. L. Sharma V. P. (2013). Reduced Dosage of ERF Causes Complex Craniosynostosis in Humans and Mice and Links ERK1/2 Signaling to Regulation of Osteogenesis. Nat. Genet. 45, 308–313. 10.1038/ng.2539 23354439 PMC3683605

[B51] Twigg S. R. F. Wilkie A. O. M. (2015). A Genetic-Pathophysiological Framework for Craniosynostosis. Am. J. Hum. Genet. 97, 359–377. 10.1016/j.ajhg.2015.07.006 26340332 PMC4564941

[B52] Ueda K. Yaoita M. Niihori T. Aoki Y. Okamoto N. (2017). Craniosynostosis in Patients with RASopathies: Accumulating Clinical Evidence for Expanding the Phenotype. Am. J. Med. Genet. 173, 2346–2352. 10.1002/ajmg.a.38337 28650561

[B53] Villa O. Del Campo M. Salido M. Gener B. Astier L. Del Valle J. (2007). Small Supernumerary Marker Chromosome Causing Partial Trisomy 6p in a Child with Craniosynostosis. Am. J. Med. Genet. 143A, 1108–1113. 10.1002/ajmg.a.31709 17431916

[B54] Wilkie A. O. M. Bochukova E. G. Hansen R. M. S. Taylor I. B. Rannan-Eliya S. V. Byren J. C. (2007). Clinical Dividends from the Molecular Genetic Diagnosis of Craniosynostosis. Am. J. Med. Genet. 143A, 1941–1949. 10.1002/ajmg.a.31905 17621648

[B55] Wilkie A. O. M. Byren J. C. Hurst J. A. Jayamohan J. Johnson D. Knight S. J. L. (2010). Prevalence and Complications of Single-Gene and Chromosomal Disorders in Craniosynostosis. Pediatrics 126, e391–e400. 10.1542/peds.2009-3491 20643727 PMC3535761

[B56] Yilmaz E. Mihci E. Nur B. Alper Ö. M. Taçoy Ş. (2019). Recent Advances in Craniosynostosis. Pediatr. Neurol. 99, 7–15. 10.1016/j.pediatrneurol.2019.01.018 31421914

[B57] Zhao M. Havrilla J. M. Fang L. Chen Y. Peng J. Liu C. (2020). Phen2Gene: Rapid Phenotype-Driven Gene Prioritization for Rare Diseases. NAR Genomics and Bioinformatics 2, lqaa032. 10.1093/nargab/lqaa032 32500119 PMC7252576

[B58] Zollino M. Lattante S. Orteschi D. Frangella S. Doronzio P. N. Contaldo I. (2017). Syndromic Craniosynostosis Can Define New Candidate Genes for Suture Development or Result from the Non-Specifc Effects of Pleiotropic Genes: Rasopathies and Chromatinopathies as Examples. Front. Neurosci. 11, 1–8. 10.3389/fnins.2017.00587 29093661 PMC5651252

