# Supplementary material for: Results from Genetic Studies in Patients Affected with Craniosynostosis: Clinical and Molecular Aspects
Source: Front Mol Biosci. 2022 Apr 28;9:865494. doi: 10.3389/fmolb.2022.865494 (PMC9112228; doi:10.3389/fmolb.2022.865494)
Supplement: Supplementary file 2 [file Table4.pdf]

Supplementary Table 4 Summary of genetic diagnosis obtained in a cohort of 166 patients affected with syndromic craniosynostosis (only patients with positive results of genetic testing were calculated)

	FGFR1		*FGFR2*		*FGFR3*		*TWIST1*		*Total*		Another		*Total*	
	**Patients**	**Frequency (%)**	**Patients**	**Frequency (%)**	**Patients**	**Frequency (%)**	**Patients**	**Frequency (%)**	**Patients**	**Frequency (%)**	**Patients**	**Frequency (%)**	**Patients**	**Frequency (%)**
**Sex**														
Female	2	3	13	19	10	14	9	13	34	48	10	14	44	62
Male	1	1	6	9	3	4	8	12	18	25	9	13	27	38
Total	3	4	19	27	13	18	17	24	52	73	19	27	71	100
**Presence of additional clinical features**														
Isolated	0	0	4	8	8	16	8	16	20	39	5	10	25	49
Syndromic	0	0	6	12	1	2	5	10	12	24	14	27	26	51
Total	0	0	10	20	9	18	13	25	32	63	19	37	51	100
**Number of affected sutures**														
Multiple	0	0	8	16	5	10	7	14	20	41	12	24	32	65
Single	0	0	1	2	4	8	5	10	10	20	7	14	17	35
Total	0	0	9	18	9	18	12	24	30	61	19	39	49	100
**Occurence**														
Familial	3	4	2	3	3	4	9	13	17	24	2	3	19	27
Sporadic	0	0	17	24	10	14	8	11	35	49	17	24	52	73
Total	3	4	19	27	13	18	17	24	52	73	19	27	71	100
