# Supplementary material for: Results from Genetic Studies in Patients Affected with Craniosynostosis: Clinical and Molecular Aspects
Source: Front Mol Biosci. 2022 Apr 28;9:865494. doi: 10.3389/fmolb.2022.865494 (PMC9112228; doi:10.3389/fmolb.2022.865494)
Supplement: Supplementary file 4 [file Table1.pdf]

Supplementary Table 1 List of primers used for PCR followed by Sanger sequencing

Gene	Exon/ variant	Forward primer sequence 5’-3’	Reverse primer sequence 5’-3’
**Confirmation studies in Tier 1**			
FGFR1	5	GGGAGGTTTACAACCCATCA	TCGATGTGCTTTAGCCACTG
FGFR2	7	GGTCTCTCATTCTCCCATCCC	CCAACAGGAAATCAAAGAACC
FGFR2	8	*tgtaaaacgacggccagt*CCTCCACAATCATTCCTGTGTC	*caggaaacagctatgacc*ATAGCAGTCAACCAAGAAAAGGG
FGFR3	7	GCGTCGTGGAGAACAAGTTT	GTGCTTGAGCCACTGGATGT
TWIST1	1	GAGGCGCCCCGCTCTTCTCC	AGCTCCTCGTAAGACTGCGGAC
TWIST1	2	CAAGAAGTCTGCGGGCTGTG	AATCGAGGTGGACTGGGAACCG
		**Confirmation studies in Tier 2 & 3**	
ALX3	c.578_581del	CCTCCCTTTTGTGTTCTCCA	ACTTCACCGCTTCCAAAATG
ARID1A	c.791C>A	ACAACATGGCGGACAACAAA	GACGTGAGCAGTTGGTTGAG
EFTUD2	c.491A>G	TTTCATTTCCTGAGGGATGC	AGGGTGAAGGAAGGAGGAGA
ERF	c.394C>T	ATTCATTGATGTGGGGTTGG	GCAGGTACCGTTTCATGTCC
FAM111A	c.913A>G	CATATTTGGCAGGCAGGACA	GTCTCCCACAATGCTATCT
KMT2A	c.93_163del	ACTTCACGGGGCGAACATG	GATGCTCCTCAATCCCGGAT
KMT2D	c.13663_13671del	GGTTGGTGAGCTCCCGAAA	CACTCTACGTCAGCAATTCCC
MN1	c.3883C>T	GGACGGTGCCTGTCTTGTAT	TCAGCAATAGTGGCCCTTTC
NSD1	c.2954_2955del	AGGAGCAGCGGTTGATGAC	CATTCTCACTGTTCATTACAGA
NSD1	c.5332C>T	TGTGGACAGACAGACATTGC	TCTGAGCCTCTGTCTAGCAA
RECQL4	c.308C>T	CTCCTCCCACTTCCCTGTTT	AGTCCCCACGCTCAATTGTA
RECQL4	c.3062G>A	CCTGCCTGCATCTGACATG	GGAGGAGCCTGTCAGAGC
TCF12	c.356T>C	TCCTGCCCAGAATTTGACTC	GCCTGGTAAACTGGGGAACT
TCF12	c.679del	GAGGTTTGTGTTTGGTAAGCC	TCAGCAATAAACACCACTGGA
TCF12	c.932C>G	ACACGTGACTAGGGTACAGC	ATCACCTCCTCAACCTTGGA
TCF12	c.2015_2016ins	CTGGAGGCTCAGTTCAATGC	ACCTTTACAATGGCCACTGC
ZIC1	c.1172C>A; c.1210T>C	GAGGCGAGTATGAGCAAAGG	TTTGATTCGCAGGGTTCTTT
